# A SCOPING REVIEW OF HOW NURSING HOME STAFFING LEVELS AND QUALITY OF CARE ARE MEASURED

**DOI:** 10.1093/geroni/igac059.2364

**Published:** 2022-12-20

**Authors:** Mutian Zhang, Shelby Brown, Weizhou Tang, Reginald Tucker-Seeley

**Affiliations:** University of Southern California, Los Angele, California, United States; University of Southern California, Los Angele, California, United States; University of Southern California, Los Angele, California, United States; University of Southern California, Los Angele, California, United States

## Abstract

The Centers for Medicare & Medicaid Services (CMS) is the largest payer of nursing home care in the US. CMS defines staffing levels as hours per resident day (HPRD) and staff turnover rates; it defines quality of care as nurse staffing levels, hospitalization rates, resident outcomes, and deficiencies (violations of regulations). However, these definitions have been inconsistently applied in the research literature, which has led to equivocal results in research on the association between staffing levels and the quality of care in nursing homes. We conducted a scoping review of how nursing home staffing levels and quality of care have been defined and measured in the research literature, guided by the PRISMA (Preferred Reporting Items for Systematically reviews and Meta-Analyses) framework. Out of the N=423 initially identified studies through PubMed, N=67 articles were selected, based upon the following inclusion criteria: 1) published after January 1, 2010; 2) published in the US; 3) focused on determinants of nursing home staffing levels or determinants of quality of care. Two independent reviewers conducted abstract screening and data extraction. The findings from our review showed that approximately 50% of studies partially adopted the definitions from CMS. For example, HPRD and deficiencies were utilized to measure staffing levels and quality of care in N=37 and N=12 studies, but none of them included all suggested measures. Future studies should carefully consider the approriate definitions as specific measures can yield contradictory associations between staffing level and quality of care, easily leading to confusion for policymakers.

